# The Effect of Dioxin Contamination and Remediation on Property Values

**DOI:** 10.3390/ijerph16203900

**Published:** 2019-10-15

**Authors:** Adam Zwickle, Jeffrey G. Cox, Jie Zhuang, Joseph A. Hamm, Brad L. Upham, Minwoong Chung, Shannon Cruz, James W. Dearing

**Affiliations:** 1 School of Criminal Justice, Michigan State University, East Lansing, MI 48824, USA; jhamm@msu.edu; 2 Environmental Science and Policy Program, Michigan State University, East Lansing, MI 48824, USA; 3 Department of Community Sustainability, Michigan State University, East Lansing, MI 48824, USA; 4 Department of Communication, Michigan State University, East Lansing, Michigan, USA; coxjeff1@msu.edu (J.G.C.); chungm12@msu.edu (M.C.); dearjim@msu.edu (J.W.D.); 5 Department of Communication Studies, 2800 South University Drive, Texas Christian University, Fort Worth, TX 76109, USA; jie.zhuang@tcu.edu; 6 Department of Pediatrics and Human Development, 1355 Bogue St, B240 Life Sciences Building, Michigan State University, East Lansing, MI 48824, USA; Brad.Upham@hc.msu.edu; 7 Communication Arts & Sciences, Pennsylvania State University, University Park, PA 16802, USA; cruzsh@oregonstate.edu

**Keywords:** dioxin, environmental contamination, remediation, property values, Midland

## Abstract

Loss of property value is a major concern in communities faced with the toxic byproducts of industrial practices. Even after site remediation, stigma may persist and negatively affect market values of residential properties. To study the effects of contamination and of remediation on property values in Midland, Michigan, where dioxins have been released into the environment through the incineration of contaminated waste and the discharge of contaminated water for many years, records of assessed value were obtained for 229 homes within the same neighborhood for the previous 18 years. A multilevel, longitudinal analysis was conducted to determine if there was a relationship between level of dioxin and assessed value after controlling for housing characteristics. Remediated and un-remediated properties saw increases in value at a similar rate over time. However, a property’s level of dioxin was found to have a small, significant, and negative relationship with assessed value, and this negative effect was present regardless if a home had been remediated or not. These results suggest that while environmental remediation may be effective at removing the contamination, its economic effects may persist for a longer period of time.

## 1. Introduction

### 1.1. The Effect of Dioxin Contamination and Remediation on Property Values

This study presents an analysis of property values in Midland, Michigan—a city that has seen considerable environmental contamination from dioxins, as well as a government-mandated response to remediate contaminated properties. Here, the term dioxin refers to all congeners of dioxin and furans that may induce harm through a similar receptor-based mechanism. As might be expected, homeowners have expressed concern about the effects of dioxin on property values. There has also been concern about the potential negative effects of the remediation process, as it requires the removal and replacement of trees, landscaping, and the top 12 inches of soil [1].

### 1.2. Environmental Contamination

Environmental contamination can have adverse economic impacts on property owners [2], yet the evidence about remediation, while suggestive, is not conclusive. Literature about the effects of environmental contamination on real estate property values generally indicates that contamination negatively affects real estate prices, but those effects tend not to persist once site remediation occurs [3]. For example, Kohlhase (1991) found that households within six miles of a toxic area in Harris County, Texas, lost value after the Environmental Protection Agency (EPA) announced that the site was listed on the National Priorities List [4]. Once the area was remediated, values rebounded. Dale, Murdoch, Thayer, and Waddell (1999) also reported that property values around an area contaminated by a smelting cooperative in Dallas County, Texas, decreased, and then rebounded when remediation was completed [5]. Finally, a meta-analysis on this issue utilizing a variety of research methodologies showed that while environmental contamination has an overall negative effect on property value, contamination from dioxin did not [6]. We conducted this study to specifically test whether this relationship exists for dioxin contamination and whether it persists past remediation.

### 1.3. Dioxins, the City of Midland, and Dow Chemical

Dioxins are a family of 75 compounds and 135 furans with similar chemical structures. Exposure to high levels of dioxins has been linked to numerous health problems, including effects on the human immune system [7]. Dioxins naturally occur in the environment in small amounts, but high concentrations of the toxicant are often the unintentional byproduct of manufacturing processes involving chlorine such as smelting, pulp and paper bleaching, combustion of chlorinated chemicals, and synthesis of chlorinated chemicals, particularly pesticides [8]. The high concentrations found in the Midland area primarily are due to manufacturing activities from the Dow Chemical Company. Decades of incinerating chlorinated chemicals and the discharge of contaminated water at Dow’s Midland facility led to the transmission of significant levels of dioxin onto properties in Midland residential areas [9]. The public has known about the presence of dioxins in the Midland area since the mid-1980s, after fish in the nearby Tittabawassee River tested with abnormally high levels of the contaminant [10,11]. While ingestion is a primary human exposure pathway, contact with contaminated soil is also considered unsafe.

Dow’s relationship with Midland residents, however, is a multifaceted one, as Dow was not only the main source of contamination, but remains the primary employer in the area. Thousands of Midland residents work for Dow or its contractors, and the company is an active investor in area schools, parks, and community centers. Its deep integration with the city and support from local residents has led Midland to be described as a “company town” [12]. Because of Dow’s complex and historic relationship with the community, public attitudes and perceptions of the company are varied [13], creating a novel research setting.

### 1.4. Testing and Remediation Process

Soil testing of certain Midland properties for elevated levels of dioxin began in 2012. An incremental composite technique was used to measure dioxin levels on residential properties, in which several samples were collected from each residential property and then combined into a single composite. If dioxin levels exceeded the site-specific action level of 250 parts per trillion (ppt), the property was scheduled for remediation. Remediation consisted of removing the top 12 inches of soil and replacing it with clean soil, a new lawn, and landscaping. Some trees and shrubs were retained on a case-by-case basis.

The reference dose of 250 ppt of dioxin for the city of Midland was based on the toxic equivalency factor established by the World Health Organization [14]. Michigan Department of Environmental Quality (MDEQ) followed Part 201 of Michigan’s Natural Resources and Environmental Protection Act (1994) to establish 250 ppt as a site-specific cleanup number using cancer endpoints for Midland [15].

### 1.5. This Study

Given the mixed literature regarding the success of remediation in removing the negative effect of contamination on property values, the unique relationship between Dow Chemical and the residents of Midland, and the highly visible process of remediation in a residential setting, we conducted this study to answer the following research questions (RQs):

RQ1: Does dioxin contamination negatively affect property values?

If so:

RQ2: Does this negative effect remain even after the contamination is remediated?

## 2. Materials and Methods

### 2.1. Data Sources and Data Preparation

Data regarding the dioxin levels of tested properties and their remediation status were obtained from a public Dow report, submitted to MDEQ in 2014. The report listed the properties which were tested in 2012 and 2013, the procedure used to collect soil samples, the dioxin levels of each property, and whether that property was remediated. We note that testing and remediation continued after 2013, but results from those tests were not made public. Our sample consisted of all available data, which included approximately 15% of the total properties that were tested and not remediated and approximately 50% of properties that were remediated [16].

Using these data, we located properties on a parcel map obtained from the Midland County Geographic Information Systems (GIS) office by matching the parcel identification numbers. This map was created by overlaying geographic data of Midland and the nearby riverfront with Midland city planning data to create a detailed picture of neighborhoods and parcel locations. Two members of the research team checked this step independently to ensure no errors.

Plotted properties were separated into two groups: those which had less than 250 ppt of dioxin (*N* = 209 parcels) and those with 250 ppt or greater (*N* = 53).

We next contacted the Midland County assessor to obtain the annual assessed value of each property over time. County and city assessors are professionally trained government officials who estimate the value of properties within a city, town, village, or county boundary annually. Each valuation is converted into an assessment, partly for the purpose of computing tax bills. Assessors make onsite inspections of properties and compare a focal property with market value of comparison properties in deriving assessed value as well as calculate the cost of replacing a house with a similar one. It is important to note that the property market value and assessed value are conceptually distinct. While the former refers to the most probable price that both a buyer and a seller would agree on a given property transaction, the latter indicates the value that assessors place on a property for tax purposes (based upon its property market value). By Michigan law, the assessed value should not exceed one-half of a given property’s market value; in practice, however, the assessed value is generally 50% of a property’s market value [17,18]. In the U.S., assessors typically have completed certification courses and gained considerable experience as property appraisers. Completion of annual continuing education is also typical.

With assistance from the Midland City Assessor’s Office, we obtained property assessment data from BS&A Software (Bath, Michigan, United States), an independent property data assessment company that partners with Michigan GIS offices. The data used in this analysis were received as a part of the ASSESSING.NET software (version 10.1.19), which included various property variables such as parcel type, school district, and home modifications. Electronic records were available from 2000–2017, and complete records for all 18 years were found for 240 of the 258 properties. Eleven of these properties were removed from the dataset since they were not zoned as residential, leaving 229 residential properties in the final study sample (see Table 1). The final breakdown of the two groups was:

- 50 properties tested, found to contain over 250 ppt of dioxin and subsequently remediated;
- 179 properties tested, found to contain levels of dioxin under the actionable level of 250 ppt and therefore not remediated.

### 2.2. Data Analysis

To determine whether contamination and/or remediation had an effect on assessed home values over time, we estimated a piecewise, longitudinal, multilevel model in which annual assessed property values for 18 years (2000–2017) were nested within each property. For this analysis we used SPSS (version 25.0) software (IBM, Armonk, New York, United States). We began with an empty means unconditional growth model in order to determine the intraclass correlation (ICC), which is the proportion of variance in assessed property values that is associated with between property factors to within properties (over time) factors. The fixed intercept, or the grand mean of the outcome variable, assessed value, over time was US$34,200. The ICC was 0.82, meaning that 82% of the total variance was found between properties, and 18% was explained by changes over time. To create a more accurate model and allow estimated property values to vary according to real world events, we separated the fixed effect of time into four time frames surrounding prominent events that may have impacted property values: A prominent and controversial town meeting, the housing market crash, and the beginning of testing and remediation (see Table 2), testing them both as linear and quadratic functions to better account for observed changes over time and entering them in the model when significant.

We used an autoregressive covariance structure to best account for the high correlation of property values year to year. Time variables were also tested as random factors, but the model only converged around the inclusion of time 2 and time 3 as random factors and not when both variables were included together. We used a change in scaled loglikelihood (−2 *LL) test to compare model fit to show that entering time 3 as a random factor resulted in better fit than time 2 (−2 ΔLL = 162), and entering time 3 as a random factor resulted in better model fit compared to a model without it (−2 ΔLL(3) = 396.1, *p* < 0.001).

We next entered the property information relevant to its value that was available: the size of house (in square feet) and size of the parcel (in acres). Both variables were mean centered (*M*: house size = 1169.07 ft^2^; lot size = 1.20 acres) and tested as a quadratic function. House size was found to have a significant, linear relationship (*b* = 0.02, *p* < 0.001), while parcel size had a significant relationship as a linear (*b* = 32.72, *p* < 0.001) and quadratic (*b* = −40.41, *p* < 0.001) term. The inclusion of each housing-related variable significantly improved the fit of the model. Combined, these time and property-related variables made up the baseline model necessary to begin our main analyses.

To determine whether contamination had an effect on the assessed value of a property, we tested two different characterizations of dioxin. As it is possible that dioxin, in parts per trillion, is simply too fine a scale, we first tested whether a property had been remediated or not had an influence on assessed home values by creating a dichotomous variable. Second, we used the actual amount of dioxin, in parts per trillion, found in the soil of each property entered as a continuous measure. Three separate models were estimated using this continuous measure, first including all homes that were tested for dioxin, then separating the sample by those that were remediated and those that did not require remediation.

## 3. Results

**Figure 1:**
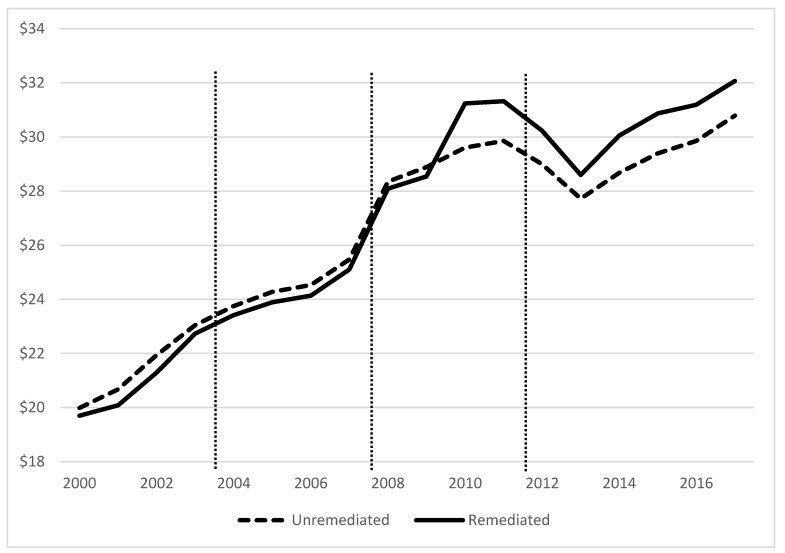
Average assessed value per square foot, separated by modeled time periods.

To begin, we visualized the data by plotting the average assessed value per square foot over time for both remediated and un-remediated properties (Figure 1). Both groups had similar relative assessed value and growth over time until just prior to the housing market crash in Midland, where the properties that would eventually be remediated continued to increase. From 2010 to the most present data, remediated homes remained assessed at a higher relative value than un-remediated homes. The testing and remediation phase, which first began in 2012, appeared to have had little effect on assessed values.

### 3.1. RQ1: Does Dioxin Contamination Negatively Affect Property Values?

In order to address our first research question, we began by testing if whether a property was remediated or not had any relation to its assessed value. The dichotomous dioxin variable had no effect (*b* = 0.56, *p* = 0.50), showing that, for those properties included in our study, there was no statistically significant difference in assessed property values between remediated and un-remediated properties (Table 3). We next tested whether the actual amount of dioxin, measured in parts per trillion, found to be present in the soil was associated with changes in assessed property values. The continuous measure of dioxin did have significant, negative effect (*b* = −0.009, *p* = 0.02, 95% CI [−0.016, −0.002]), such that for every 1 ppt increase of dioxin, the predicted assessed value of an average sized home decreased US $9.

### 3.2. RQ2: Does this Negative Effect Remain Even After the Contamination is Remediated?

To answer our second question, we first obtained separate fixed effects of dioxin for each group of properties and then tested for an interaction between level of dioxin and remediation status. For this model, we calculated the mean level of dioxin for each group and then centered each property’s dioxin level around their group mean. We then entered that group mean centered level of dioxin, a remediation dummy code (un-remediated, remediated), and the interaction between the two into the longitudinal, multilevel model.

Dioxin was found to have a significant, negative effect on assessed property values for those homes which were under the actionable limit and therefore not remediated (*b* = −0.027, *p* < 0.05, 95% CI [−0.049, −0.006]), such that for every 1 ppt increase of dioxin, the predicted assessed value of an average sized home not receiving remediation decreased US $27. The effect for dioxin on remediated homes was not significant (*b* = −0.008, *p* = 0.16).

The property group dummy code (*b* = 0.81, *p* = 0.34) and the interaction between dioxin and the property group (*b* = −0.02, *p* = 0.13) were both insignificant, suggesting that, while dioxin did have a negative relationship for un-remediated homes but not for remediated ones, the difference between those relationships was not significant.

Finally, we conducted an additional test to directly assess the robustness of the effect of dioxin first on all properties, and then on both un-remediated and remediated properties separately. The effect size of dioxin was determined by standardizing assessed value, house size, lot size, and dioxin and running the model as before. The effect size of dioxin on assessed value was consistently small, with a standardized beta coefficient of −0.13 for the overall sample (*p* < 0.001), −0.09 for un-remediated properties (*p* < 0.05), and −0.07 for remediated properties (*p* = 0.21).

## 4. Discussion

### 4.1. General

This study evaluated the assessed value for properties in Midland, Michigan, that were tested for dioxin contamination to determine if this contamination and subsequent remediation had an impact on assessed property values. We linked the amount of dioxin to 18 years of assessment records to conduct a piecewise, longitudinal, multilevel model. When using a dichotomous measure of dioxin (whether or not a property was over the actionable limit and subsequently remediated), no significant relationship with assessed property values was found. However, when we substituted a higher resolution measure of dioxin (the actual ppt amount of dioxin found in a soil test), there was an overall negative relationship with assessed property values. A more parsimonious test showed this negative effect to be significant for un-remediated homes but not for those homes which had the dioxin contamination removed. Further analysis revealed that this relationship was a weak one and did not significantly differ based on remediation status. Given this, it is possible the lack of relationship between dioxin and remediated home assessed values is a result of the small effect size coupled with smaller sample size (*n* = 50). The presence of the negative relationship between assessed value and dioxin should be interpreted with caution, as standardizing the model variables showed this to be a small effect. That being said, this relationship is significant and present in the overall sample. While the remediation process is effective at removing the environmental contamination, it does not appear to remove its negative impact on a home’s assessed value. These results suggest that dioxin can have a similar financial impact on property values as other contaminates [6]. Another interpretation of this finding is that the remediation process itself is not associated with a decrease in assessed value. Given the invasive nature of remediation, removal of the top 12 inches of soil and all vegetation, the nonsignificant effects of the dichotomous dioxin variable and the interaction term suggest that the environmental contaminants can be removed without causing further economic harm. This speaks to some concerns from the community; while some residents were pleased to receive new landscaping, others lamented the loss of their original vegetation.

In regards to our original research questions, our data suggest that higher levels of dioxin are, in fact, associated with lower values in these properties (RQ1) and that this negative effect is present for both remediated and un-remediated properties (RQ2). In addition, this study provides additional evidence in support of using remediation to address the negative environmental impacts of contamination without inflicting further economic harm.

### 4.2. Limitations

There are several important limitations in this study. First, and most importantly, our data are not able to explain the increase in assessed home values from 2009–2010 among those homes that would eventually be remediated relative to those that would not. This was the period of time when the subprime mortgage crisis was occurring in the United States, but before its effects had hit the housing market in Midland. An attempt to compare home sales over time in Midland was unsuccessful because of the issues of sample size and availability of data. Likewise, we could not compare trends in home sales between Midland to the rest of Michigan because of the unique nature of the city’s small, highly educated, and stable population. It is counterintuitive to expect home values to increase because of contamination, and, to our knowledge, contamination data were not publicly available at this time. Yet this increase differs across groups and occurs after controlling for house and lot size among homes located in the same neighborhood. Follow-up research should seek to understand this increase better, as this gap in home values remains until the end of our study period.

Second, our data did not include other variables that could have an impact on assessed value, namely variables associated with the location and age of properties. We believe the exclusion of location variables to be less of an issue, as all of the homes in this study were located in close proximity to each other and within the same school district. Midland is a small community, and the study area comprises approximately one square mile. Within this area, the distribution of dioxin, and therefore the pattern of remediation, appears to be random. Regarding the age of properties, it is possible that both contamination and assessed value could be related to the construction date of a home. If contamination did vary by age of home, however, the variation in age of homes is relatively small, as the study area excludes newer developments located further away from Dow’s facilities.

Third, the dependent variable used was the assessed value of a property, not its actual selling value. Although assessed values may differ systematically from a home’s selling value, we considered the benefits of its use as a proxy measure outweighed the risks. The use of assessed value as a proxy measure enabled us to significantly increase the number of data points in our sample, as we were able to include every home for each year that we had data. Additionally, while county and city assessors in Michigan are mandated to keep assessed values no greater than 50% of the projected sale value, in practice they are typically set right at that cutoff. Therefore, the values used in these analyses, while not reflective of the sale price, should be consistently proportional to it. Furthermore, all properties for the duration of this study were assessed in a consistent manner by the same assessor, removing the risk of error associated with differences in personnel.

Finally, while our analysis used all of the data made publicly available, the missing data cannot be considered missing at random. The data used here came from the first two phases of a three-phase testing and remediation process, and the decision criteria used to determine where cleanup began are not known. While the first two phases contain most homes within the city limits, the justification for the boundaries of each phase are not known. At any rate, as properties in the third phase differ in location, size, and zoning from those in our sample, many of them would have been excluded by our study design, which focused only on residential properties within the same school district.

## 5. Conclusions

Our study into the impacts of dioxin contamination and subsequent remediation in Midland, Michigan, found that dioxin had a significant, negative effect on assessed property values. This effect did not differ between those properties that had been remediated to remove the contamination and those that were below the actionable limit and therefore not remediated.

## Figures and Tables

**Table 1:** Descriptive statistics for sampled homes.

	Un-Remediated Homes	Remediated Homes
*N*	179	50
Average assessed value (USD)		
2000	$23,463 ± $9816	$19,982 ± $4811
2017	$35,640 ± $10,177	$32,466 ± $7148
Average house size (ft^2^)	1202.60 ± 429.91	1049.04 ± 293.46
Average lot size (acres)	0.20 ± 0.17	0.18 ± 0.11
Average amount of dioxin (ppt)	173.35 ± 39.50	332.40 ± 135.55

**Table 2:** Piecemeal time line variables and the corresponding event at the onset.

	Years	Corresponding Event
Time 1	2000–2004	Beginning of electronic assessment records
Time 2	2005–2008	Contentious public meeting
Time 3	2009–2013	Beginning of housing downturn in Michigan
Time 4	2013–2017	Beginning of testing and remediation

**Table 3:** Fixed effect estimates from longitudinal, multilevel analyses of the effect of dioxin on assessed home values, in thousands of US dollars. (*n* = 229).

Variable		Dioxin Characterization		
	Base Model	Dichotomous	Continuous	Group Mean Centered
Intercept	23.61	23.17	25.53	23.13
Piecemeal time variables				
Time 1	0.96	0.96	0.96	0.96
Time 2	ns	ns	ns	ns
Time 2^2^	0.18	0.18	0.18	0.18
Time 3	−0.78 ^†^	−0.78 ^†^	−0.84	−0.83
Time 3^2^	−0.58	−0.58	−0.59	−0.59
Time 4	3.00	3.00	3.01	3.01
Time 4^2^	0.36	0.36	0.36	0.36
Property variables				
House size (ft^2^)	0.02	0.02	0.02	0.02
Lot size (acres)	32.72	32.242	35.48	35.94
Lot size^2^	−40.41	−40.495	−46.43	−47.61
Dioxin variables				
Dichotomous (remediated/un-remediated)		ns		
Continuous (ppt)			−0.009 *	
Group Mean Centered				
Un-remediated homes				−0.027 *
Remediated homes				ns
Interaction (group*dioxin)				ns
–2 log likelihood	19,300.37	19,299.92	19,261.60	19,258.14

Note: All estimates significant at *p* < 0.001 unless otherwise noted, * *p* < 0.05, ^†^ *p* < 0.01; ns: not significant.
